# Infective endocarditis mimicking antineutrophil-cytoplasmic-antibody-associated vasculitis with glomerulonephritis: a case report

**DOI:** 10.1186/s13256-025-05470-1

**Published:** 2025-08-04

**Authors:** Ahmad Matarneh, Sundus Sardar, Abdelrauof Akkari, Omar Salameh, Naman Trivedi, Muhammad Abdulbasit, Navin Verma, Ronald Miller, Nasrollah Ghahramani

**Affiliations:** 1 Department of Nephrology, Penn State Health Milton S Hershey Medical Center, Hershey, USA; 2 Department of Internal Medicine, Penn State Milton S Hershey Medical Center, Hershey, USA

**Keywords:** ANCA vasculitis, Autoimmune disease, Infective endocarditis, Immune suppression

## Abstract

**Background:**

Infective endocarditis occasionally presents with antineutrophil cytoplasmic antibody positivity, leading to diagnostic challenges and confusion, as it can be mislabeled antineutrophil-cytoplasmic-antibody-associated vasculitis. Distinguishing between these two factors is crucial for appropriate management.

**Case presentation:**

In this case report, we describe a 77-year-old White non-Hispanic male patient who initially presented with features suggestive of antineutrophil-cytoplasmic-antibody-associated vasculitis but was ultimately diagnosed with infective endocarditis.

**Conclusion:**

Our findings emphasize the need to rule out infective endocarditis in patients with suspected antineutrophil-cytoplasmic-antibody-associated vasculitis, as it can be the same, and management relies on different lines of therapy. Immunosuppression therapy can lead to devastating effects in patients with infective endocarditis.

## Background

Infective endocarditis (IE) is a rare and serious infection characterized by microbial invasion of cardiac valves and/or the endocardium. IE can present with a wide spectrum of clinical manifestations, including renal involvement and immune-mediated phenomena such as Osler nodes and glomerulonephritis [1]. Diagnosis can lead to significant morbidity and mortality if the disease is not diagnosed and treated in a timely manner. IE can sometimes be associated with antineutrophil cytoplasmic antibody (ANCA) positivity; the incidence is not clear, but it has been postulated to be around 18–24% in some studies [2]. ANCA-associated vasculitis comprises a group of autoimmune disorders characterized by inflammation and necrosis of small- to medium-sized blood vessels, often affecting the kidneys, lungs, and other organs. The presence of ANCAs in the context of IE poses significant diagnostic challenges, as it may lead to the misdiagnosis of IE as primary ANCA-associated vasculitis (AAV) or vice versa. Both conditions can present with constitutional symptoms such as fever, weight loss, and loss of appetite. However, physical exam findings can aid differentiation: palpable purpura is more typical of AAV, while Osler nodes and Janeway lesions suggest IE due to embolic phenomena [3].

Patients with IE and ANCA positivity may present with symptoms and laboratory abnormalities suggestive of systemic vasculitis, such as fever, arthralgia, glomerulonephritis, and pulmonary involvement. Additionally, kidney biopsy findings in these patients may reveal a spectrum of renal pathologies, ranging from immune-complex-mediated glomerulonephritis to pauci-immune necrotizing glomerulonephritis, further blurring the lines between infectious and autoimmune etiologies [4].

Given the potentially severe consequences of misdiagnosis and inappropriate treatment, accurate differentiation between IE with ANCA positivity and primary AAV is paramount [5]. We report a patient with IE who had a kidney injury and was found to have high ANCA titers. Kidney biopsy revealed crescentic glomerulonephritis. However, blood cultures and transthoracic echocardiogram (TTE) confirmed the presence of IE.

## Case presentation

A 77-year-old White non-Hispanic male with a known history of chronic kidney disease stage 3b presented with complaints of persistent fatigue, fever, and generalized malaise. His previous medical history included hypertension controlled on amlodipine. Upon examination, the patient had normal blood pressure and normal chest and cardiovascular examinations, and the results of the rest of the systems examination were unremarkable. Laboratory investigations revealed anemia (hemoglobin [Hb] 10.1 gm/dL), thrombocytopenia (platelets [Plt] 93 × 10^3^/ μL), and a mild elevation in creatinine levels from baseline (2.69 gm/dL from 2.53 gm/dL), indicating acute kidney injury. A comprehensive autoimmune workup was initiated, revealing the following findings: low C3 levels, normal C4 levels, and the presence of ANCA antibodies, particularly PR-3, at a titer of 50. Given the complexity of his presentation, a kidney biopsy was performed, revealing pathological findings consistent with crescentic glomerulonephritis (Table 1).

**Table 1:** Laboratory results of the patient

Lab value	Reference value	On admission	After 5 days of presentation
Hb	12–14 g/dL	10.1	9.5
Platelets	150–250 × 10^3^/μL	93	70
WBC	4–10 10^3^/μL	7.7	8.1
Creatinine	0.7–1.3 mg/dL	2.69	2
C3	90–190 mg/dL	70	—
C4	10–40 mg/dL	22	—
ANCA	—	Not detectable	—
Anti-PR3	—	47	—
Anti-MPO	—	0	
ANA	Negative	1:1280	—
Albumin	3.5–4 mg/dL	2.8	1.6
CRP	< 5	11.5	10.8
Urine WBC	0–4	0–4	10
Urine RBC	0–4	30–34	20–29
Urine protein	Negative	+30	+100

CRP, C-reactive protein; RBC, red blood cells; WBC, white blood cells

**Fig. 1:**
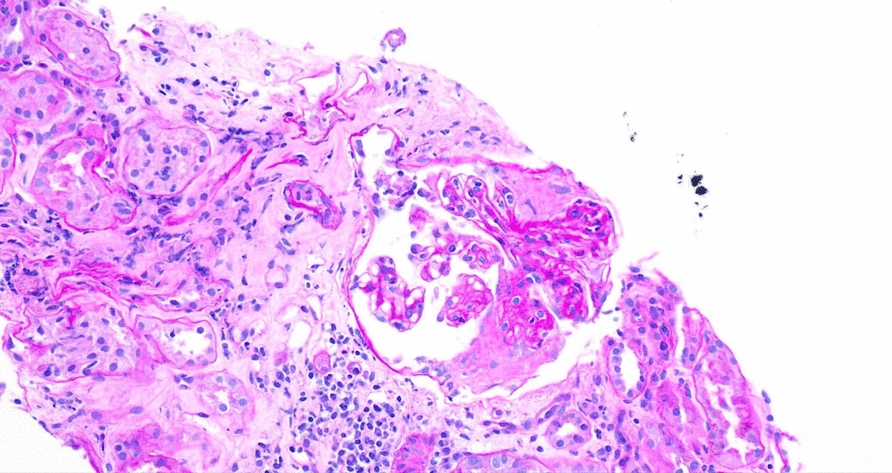
Light microscopy showing fibrous crescent and segmental sclerosis

**Fig. 2:**
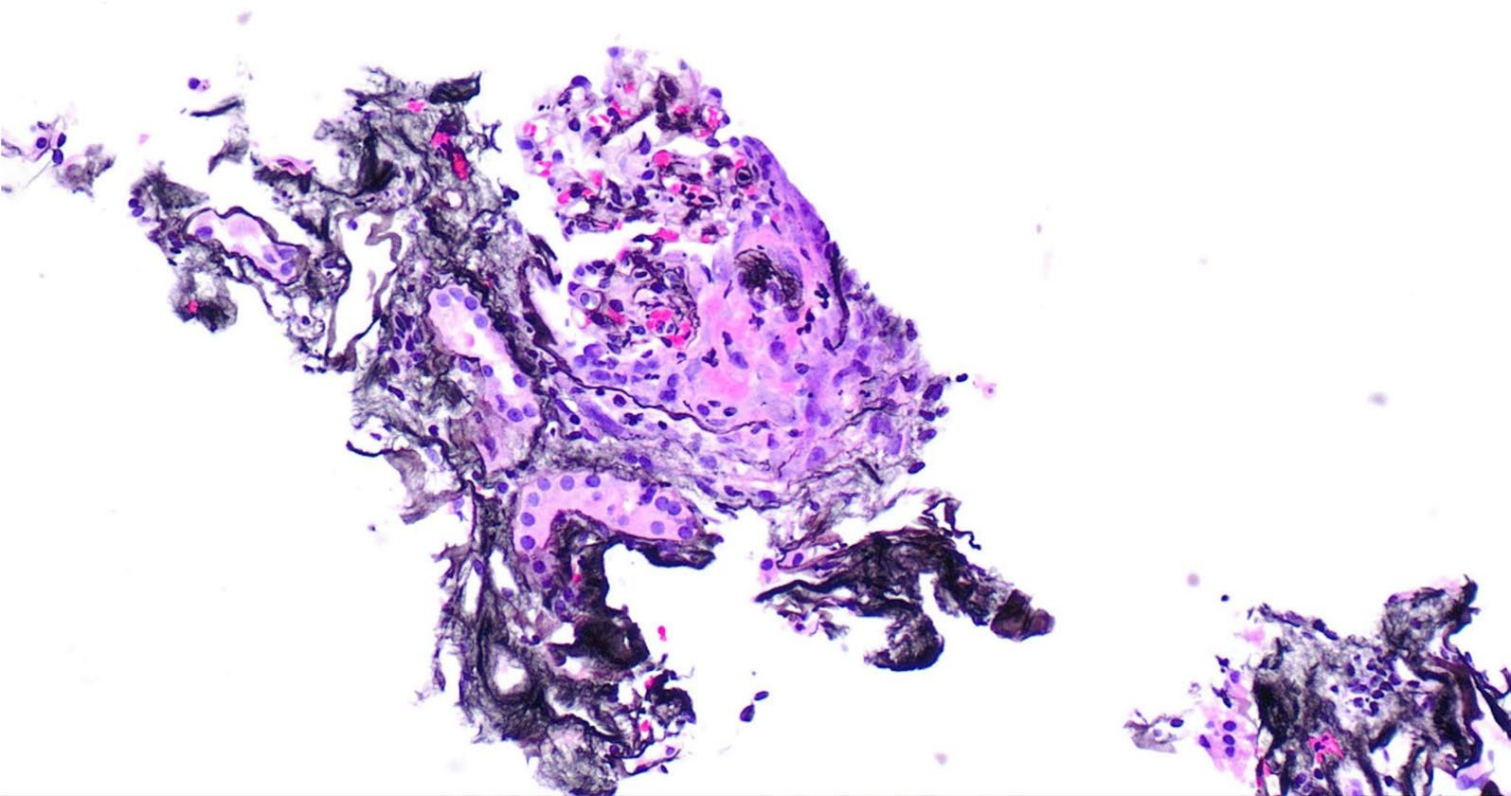
Light microscopy showing fibrous crescent

**Fig. 3:**
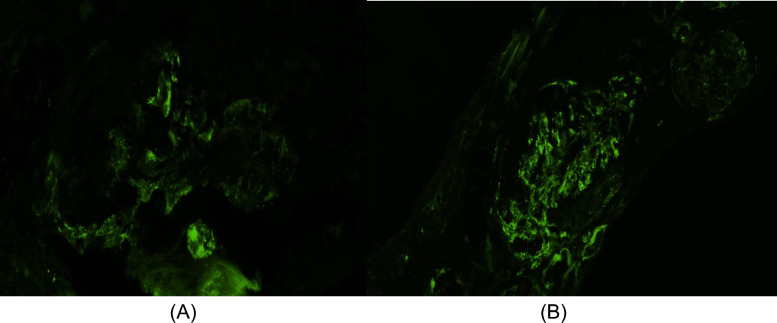
Immunofluorescence showing diffuse IgM (**A**) and C3 (**B**) deposits within the mesangium

The biopsy results revealed evidence of severe inflammation and renal injury, with crescents observed via light microscopy and an array of immune complex deposits noted via immunofluorescence, including C3, IgM, IgA, C1q, and albumin. However, electron microscopy could not provide further insights, because of the absence of intact glomeruli for examination. Immunosuppression was recommended, but the patient opted to seek a second opinion, leading to his transfer to our hospital (Figs. 1, 2 and 3).

Shortly after hospitalization, his creatinine levels began to normalize with conservative management. A thorough investigation of potential underlying infectious triggers was conducted. He received one dose of intravenous methylprednisolone 100 mg once a day, with a plan to continue for a total of 3 days and then transition to oral prednisone 60 mg once a day. The patient tested positive for coronavirus disease 2019 (COVID-19), and blood cultures revealed the growth of *Streptococcus viridans*. Unfortunately, it was difficult to test for susceptibility, indicating possible concomitant bacterial infection. Further evaluation, including echocardiography, revealed severe aortic regurgitation secondary to aortic valve endocarditis, accompanied by leaflet perforation and sizable vegetation on both the aortic and mitral valves. The patient was subsequently started on antibiotics for infective endocarditis. He was given ceftriaxone 2 g once a day and vancomycin 1500 mg as loading with further doses adjusted according to trough level. Given the overall presentation, the likely cause of the positive ANCA antibodies was related to infective endocarditis itself rather than an autoimmune process. Immunosuppressive therapy was discontinued, and antibiotics were continued. His kidney function remained stable throughout the hospital stay. The cardiothoracic surgery team was involved, and the patient underwent aortic valve replacement. Unfortunately, the patient’s postoperative course was complicated and marked by the development of cardiogenic and septic shock, prompting aggressive interventions such as mechanical ventilation, circulatory support with vasopressor therapy, a left ventricular assist device, and continuous renal replacement therapy for acute kidney injury; his second acute kidney injury was considered a part of acute tubular necrosis. Despite the concerted efforts of the medical team, his clinical status continued to deteriorate, and and he ultimately passed away.

## Discussion

This case describes the clinical overlap and diagnostic challenges encountered when IE presents with ANCA positivity, mimicking ANCA-associated vasculitis (AAV). IE typically manifests with fever, fatigue, and various systemic manifestations, whereas AAV primarily affects small blood vessels, leading to organ damage [6]. However, the presence of ANCA antibodies in IE can complicate the diagnostic process, as observed in our patient. ANCA-associated vasculitis is a group of primary autoimmune disorders; however, ANCA positivity can still occur in other conditions, such as drug-induced vasculitis and infection-associated vasculitis [7]. In addition to infection-associated vasculitis, ANCA positivity has been well-documented in inflammatory bowel disease (IBD), particularly in ulcerative colitis (UC), where perinuclear ANCA (p-ANCA) is detected in up to 60–80% of cases. Although ANCA positivity in IBD is not typically associated with active vasculitis, severe disease flares can occasionally mimic systemic vasculitis, presenting with glomerulonephritis, skin vasculitis, or pulmonary involvement [8]. This overlap further complicates the diagnostic process, especially in critically ill patients.

Similarly, chronic infections such as infective endocarditis (IE) can induce ANCA formation through persistent immune activation, as described in several case reports [9]. Furthermore, drug-induced vasculitis triggered by medications such as hydralazine, minocycline, or propylthiouracil can also cause ANCA positivity with systemic features [10]. These diverse clinical presentations highlight the importance of interpreting ANCA results within the broader clinical context, supported by microbiological cultures, imaging studies, and, when feasible, histopathological evaluation to distinguish between infectious and autoimmune causes of vasculitis.

The standard therapy for ANCA vasculitis is immunosuppression with steroids and other immunosuppressive agents; however, if immune suppression is used in cases of infection associated with vasculitis, it can lead to catastrophic outcomes [11]. Therefore, it is essential to establish an accurate diagnosis and rule out infection as a cause of ANCA positivity prior to initiating immunosuppression therapy. The clinical management of such cases requires careful consideration of the underlying pathology. While immunosuppressive therapy is the cornerstone of treatment for AAV, inappropriate use of immunosuppressants in IE can exacerbate infection and lead to disastrous outcomes [12]. Therefore, distinguishing between IE and AAV is paramount for effectively guiding therapy. Histopathological examination plays a crucial role in differentiating between IE and AAV. Kidney biopsy findings often reveal a spectrum of changes reflective of immune complex deposition and vasculitis [13]. Histopathological examination typically reveals features of pauci-immune glomerulonephritis, characterized by segmental necrosis and crescent formation in glomeruli, along with interstitial inflammation and tubular injury. Immunofluorescence studies might reveal minimal or no immune complex deposition, which is consistent with the pauci-immune pattern [14]. Additionally, vascular changes such as arteritis and thrombotic microangiopathy may be present, contributing to renal dysfunction [15]. In our patient, the biopsy revealed immune-complex deposition suggestive of glomerulonephritis, along with pauci-immune features, which are characteristic of AAV.

Differentiating between infective endocarditis (IE) and ANCA-associated vasculitis (AAV) is crucial, as initiating immunosuppressive therapy in IE can have catastrophic consequences. Therefore, it is essential to rule out infectious causes that mimic pauci-immune glomerulonephritis.

Our patient received only 1 day of steroids. Due to a high index of suspicion and prompt recognition of possible ANCA-positive infective endocarditis (IE), steroids were discontinued, reducing the risks associated with immunosuppression. In a study of 130 patients with ANCA-positive IE, 16% died regardless of treatment approach. Kidney function was restored to baseline in 31% of cases, with slightly better outcomes in those treated with antibiotics alone (37%). However, 18% showed no improvement, highlighting the complexity of managing this challenging condition [16].

## Conclusions

This case demonstrates the challenges posed by infective endocarditis (IE) presenting with antineutrophil cytoplasmic antibody (ANCA) positivity, which mimics ANCA-associated vasculitis (AAV). Through a complete evaluation of the patient’s clinical presentation, laboratory findings, and histopathological examination, we were able to distinguish between the two conditions and guide appropriate management. Our case highlights the importance of maintaining a high index of suspicion for IE in the presence of ANCA positivity to avoid inappropriate immunosuppressive therapy. By emphasizing the importance of early recognition and tailored management strategies, we can optimize outcomes and prevent potential harm to patients with similar presentations in the future.

## Data Availability

The datasets used and/or analyzed during the current study are available from the corresponding author on reasonable request.
